# Screening for tuberculosis and the use of a borderline zone for the interpretation of the interferon-γ release assay (IGRA) in Portuguese healthcare workers

**DOI:** 10.1186/1745-6673-8-1

**Published:** 2013-01-28

**Authors:** Albert Nienhaus, José Torres Costa

**Affiliations:** 1 University Medical Center Hamburg-Eppendorf, Institute for Health Services Research in Dermatology and Nursing, Martinistrasse 52, 20246, Hamburg, Germany; 2 Institution for Statutory Accident Insurance and Prevention in the Health and Welfare Services, Hamburg, Germany; 3 Faculty of Medicine, Porto University, Alameda Professor Hernâni Monteiro, Porto, Portugal

**Keywords:** Interferon-γ release assay, Tuberculosis, Healthcare workers

## Abstract

**Introduction:**

The effect of using a borderline zone for the interpretation of the interferon-γ release assay (IGRA) on the prediction of progression to active tuberculosis (TB) in healthcare workers (HCW) is analysed.

**Methods:**

Data from a published study on TB screening in Portuguese HCW is reanalysed using a borderline zone for the interpretation of the IGRA. Testing was performed with the QuantiFERON-TB Gold In-Tube (QFT). The borderline zone for the QFT was defined as interferon (INF) in QFT ≥0.2 to <0.7 IU/mL. An X-ray was performed when the IGRA was positive (≥0.35 IU/mL) or typical symptoms were present. Sputum analysis was performed according to the X-ray or the presence of typical symptoms.

**Results:**

The cohort comprised 2,884 HCW with a QFT that could be interpreted. In 1,780 (61.7%) HCW, the QFT was <0.2 IU/mL. A borderline result was found in 341 (11.8%) and a QFT >0.7 IU/mL in 763 (26.3%) HCW. Fifty-seven HCW had a TB in their medical history, eight had a TB at the time of screening and progression to active TB was observed in four HCW. Two out of eight HCW (25%) with active TB at the time of screening had a QFT result falling into the borderline zone. One out of four HCW (25%) who progressed towards active TB after being tested with QFT had QFT results falling into the borderline zone. A second IGRA was performed in 1,199 HCW. In total, 292 (24.4%) HCW had at least one of the two IGRA results pertaining to the borderline zone.

**Conclusion:**

Using a borderline zone for the QFT from 0.2 to 0.7 IU/mL should be administered with care, as active TB as well as progression to active TB might be overlooked. Therefore, the borderline zone should be restricted to populations with a low TB risk only.

## Introduction

Screening healthcare workers (HCW) for latent tuberculosis infection (LTBI) and active tuberculosis (TB) is fundamental in infection control programmes in hospitals [1]. Meanwhile, the interferon-γ release assay (IGRA) is widely used for TB screening in HCW [2-10]. Nevertheless, some questions concerning the interpretation of the results of the IGRA remain unanswered. This is particularly true for the interpretation of the IGRA in the serial testing of HCW.

Three reviews have covered the topic of IGRA variability in the serial testing of HCW so far [11-13]. All three came to the conclusion that the reversion of positive IGRA results to negative results occurs more often than conversion from negative to positive IGRA results. And more importantly, the probability of conversion or reversion depends on the quantitative results of the first IGRA. Therefore, a borderline zone might be helpful in order to separate real conversions and reversions in IGRA from variation caused by chance.

Two IGRA are commercially available: the ELISA-based QuantiFERON®-TB Gold In-Tube (QFT) and the ELISPOT-based T-SPOT.TB®. So far, a borderline zone is recommended for the T-SPOT.TB by the Centers for Disease Control and Prevention (CDC) as well as the European Centers for Disease Control and Prevention (ECDC) [14,15]. However, this recommendation is based on two rather small studies [16,17] and so far no consensus has been reached regarding the definition of such a borderline zone for the QFT.

Therefore, we reanalysed our data concerning TB and progression towards active TB in HCW screened with the QFT [18], using a borderline zone from 0.2 to <0.7 IU/mL for the specific INF-γ release.

## Methods

All workers at the Hospital São João are offered TB screening according to guidelines from the CDC [1]. Upon starting employment, all workers are examined to exclude active TB and to assess their pre-employment status. Depending on the risk assessment, the examination is repeated annually or every other year. HCW with close contact to patients in the infection and TB wards are considered to be at a high risk, workers with regular contact to patients in the other wards are considered to be at a medium risk and workers with no regular patient contact and no contact with biological material are considered to be at a low risk. After unprotected contact with an infectious patient, co-worker or material, screening is performed as well.

Since January 2007, screening has been performed using the IGRA. A chest X-ray is performed in order to exclude active pulmonary disease when the IGRA is positive and in HCW with symptoms. For the IGRA, the QuantiFERON-TB® Gold In-Tube assay (Cellestis Limited, Carnegie, Australia) is used. This whole-blood assay uses overlapping peptides corresponding to ESAT-6, CFP-10 and a portion of the tuberculosis antigen TB7.7 (Rv2654). Stimulation of the antigenic mixture occurs within the tube used to collect blood. The tubes were incubated overnight at 37°C before centrifugation, and INF-γ release is measured by ELISA according to the manufacturer’s protocol. All assays performed met the manufacturer’s quality control standards. The test is considered positive when INF-γ is ≥0.35 IU/mL after correction for the negative control.

For this analysis, a borderline zone from 0.2 to <0.7 IU/mL was assumed, as proposed by a multicentre analysis of serial QFT testing in HCW [19]. Therefore, a QFT result of <0.2 IU/mL was considered negative, a result of 0.2 to <0.7 IU/mL was considered borderline and a result of ≥0.7 IU/mL was considered positive.

Data analysis was performed using SPSS, Version 14 (SPSS Inc., Chicago, Illinois). All persons gave their informed consent prior to their inclusion in the study. No additional data was collected for the purposes of the study and the analysis was performed using anonymous data. Therefore, no endorsement by an ethics committee was required.

## Results

**Figure 1:**
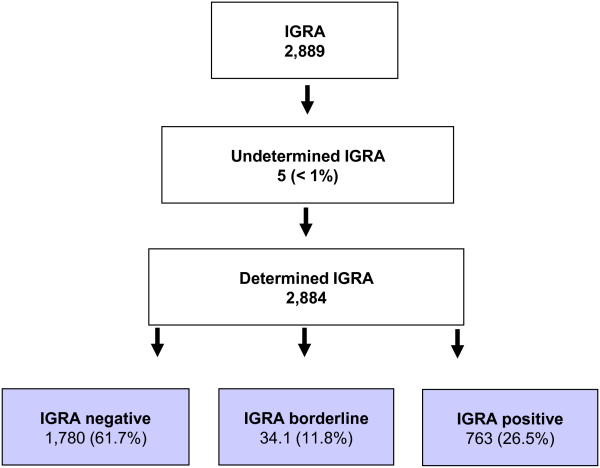
Flow chart of study population.

The study population comprises 2,885 HCW (Figure 1). The IGRA was indeterminate in five HCW. 61.7% of the HCW had a QFT result below the borderline zone (<0.2 IU/mL). 11.8% had a result falling into the borderline zone and 25.9% of the HCW tested had a positive QFT (≥0.7 IU/mL). Fifty-seven (2.0%) HCW had a history of active TB. Active TB was diagnosed in eight HCW during screening and four HCW showed progression towards active TB after a positive IGRA. Out of the 57 HCW with an active TB in their history, 16 (28.1%) had a negative IGRA and 15 (26.3%) had an IGRA result that pertains to the borderline zone (Table 1). None of the HCW with a negative IGRA result was diagnosed with active TB during screening, or developed active TB during the follow-up period. Two HCW out of eight (25%) with active TB at the time of screening had IGRA results falling into the borderline zone. One HCW out of four (25%) who progressed towards active TB after a positive IGRA result in the screening had an IGRA result that belongs in the borderline zone. Therefore, applying a borderline zone would have rendered the diagnosis of TB more difficult in three (25%) HCW out of twelve in whom active TB was observed.

**Table 1:** Results of IGRA and TB in HCW

**TB**	**QFT results**						**Total**	
	**Negative**		**Borderline**		**Positive**			
	**<0.2 IU/mL**		**0.2– <0.7 IU/mL**		**≥0.7 IU/mL**			
	**n**	**%**	**N**	**%**	**n**	**%**	**n**	**%**
TB in history	16	28.1	15	26.3	26	45.6	57	2.0
Active TB at screening	0	-	2	25.0	6	75.0	8	0.3
Progression to active TB	0	-	1	25.0	3	75.0	4	0.1
No TB	1,764	62.7	323	11.5	728	25.9	2,815	97.6
All	1,780	61.7	341	11.8	763	26.5	2,884	100.0

A second IGRA was performed in 1,199 HCW. The average time span between the two IGRA was 13.5 months (range: 0–44 months, standard deviation: 8.5 months). The result of the first IGRA was <0.2 IU/mL in 717 (59.8%), and the result of the second IGRA was <0.2 IU/mL in 705 (58.8%) HCW (Table 2). The number of HCW with a positive IGRA (≥0.7 IU/mL) increased from 309 (25.7%) in the first IGRA to 336 (28.0%) in the second IGRA. In total, 292 (24.4%) HCW had at least one of the two IGRA results pertaining to the borderline zone (calculated from Table 2). A conversion defined as trespassing of the borderline zone from negative to positive was observed in 56 (7.8%) of the HCW with a negative (<0.2 IU/mL) first IGRA. A reversion analogous to this definition was observed in 45 (14.6%) of the HCW with a first IGRA result of ≥0.7 IU/mL. With a simple dichotomous approach, conversion and reversion rates were about twice as high at 16.3% and 29.0%, respectively (Table 3).

**Table 2:** Conversion and reversion rates depending on the use of a borderline zone and the INF-γ concentration of the first QFT

**First QFT**	**Second QFT**						**Total**	
	**<0.2 IU/mL**		**0.2– <0.7 IU/mL**		**≥0.7 IU/mL**			
	**n**	**%**	**n**	**%**	**n**	**%**	**n**	**%**
<0.2 IU/mL	588	82.0	73	10.2	56	7.8	717	59.8
0.2– < 0.35 IU/mL	24	35.3	18	26.5	26	38.2	68	5.7
0.35– < 0.7 IU/mL	48	45.7	21	20.0	36	34.3	105	8.8
≥0.7 IU/mL	45	14.6	46	14.9	218	70.6	309	25.8
All	705	58.8	158	13.2	336	28.0	1199	100.0

**Table 3:** Results of the first and second QFT (negative: <0.35 IU/mL, positive: ≥0.35 IU/mL)

**First QFT**	**Second QFT**				**Total**	
	**Negative**		**Positive**			
	**n**	**%**	**n**	**%**	**n**	**%**
Negative	657	83.7	128	16.3	785	
Positive	120	29.0	294	71.0	414	
All	777	64.8	422	35.2	1,199	100.0

With the time between the two IGRA the probability of a conversion (trespassing the borderline zone) increased (Table 4). However, the increase was not monotonous. With less than nine months between the two IGRA, the conversion rate was 6.0%, with nine to 16 months in between it was 4.1% and with ≥16 months it was 14.4% (Table 4). The reversion rate did decrease with time from 30.6% to 3.8% and 7.5%, respectively.

**Table 4:** Conversion and reversion rates depending on the use of a borderline zone and the time span between the first and second QFT

**Time between first and second QFT**	**Second QFT**						**Total**	
	**<0.2 IU/mL**		**0.2– <0.7 IU/mL**		**≥0.7 IU/mL**			
	**n**	**%**	**n**	**%**	**n**	**%**	**n**	**%**
First QFT <0.2 IU/mL								
<9 months	167	83.1	22	10.9	12	6.0	201	100.0
9– < 16 months	263	89.5	19	6.5	12	4.1	294	100.0
≥16 months	158	71.2	32	14.4	32	14.4	222	100.0
First QFT ≥0.7 IU/mL								
<9 months	34	30.6	15	13.5	62	55.9	111	100.0
9– < 16 months	4	3.8	16	15.2	85	81.0	105	100.0
≥16 months	7	7.5	15	16.1	71	76.3	93	100.0

## Discussion

This is the first study to report data on disease detection in TB screenings of HCW and on disease prediction with the IGRA using a borderline zone for the interpretation of the QFT. Applying a borderline zone, the conversion was reduced to 7.8%, which seems to be more realistic than the 14.6% with a dichotomous approach, taking the relatively short average time span between the two IGRA into consideration (mean: 13.5 months). The reversion rate is reduced from 29.0% to 14.6%. Assuming that a latent TB infection (LTBI) is long lasting and immunologic responses to the LTBI do not change rapidly, the lower reversion rate might be more realistic, too.

However, introducing a borderline zone comes with a price. The QFT is less sensitive for active TB and for the prediction of TB when a borderline zone is applied and a chest X-ray is not performed in those with a QFT result within the borderline zone. In our population in particular, 25% of the active TB cases detected would probably have suffered from delayed diagnosis when strictly applying the borderline zone. As the positive predictive value of a laboratory test increases with the prevalence of the diseases tested for and vice versa, it might be safe to assume that in a population with a very low prevalence, and therefore incidence, of active TB, the application of a borderline zone is beneficial. Under these circumstances, the number of X-rays and preventive chemotherapies is reduced, rendering the screening to likely be more efficient. The HCW studies from the United States published recently might describe typical cohorts that will profit from the introduction of a borderline zone for QFT. In these studies, the rates of positive QFT were low and reversion rates were high [20-22]. In parenthesis, it might be considered if TB screening in these populations is efficient at all. In a population with a higher risk of TB, the introduction of a borderline zone for the QFT may be harmful, as was shown by our data.

To our dismay, we are not able to propose which TB prevalence the use of a borderline zone for the QFT might be useful for. Until today, data on disease detection in TB screenings of HCW and on disease prediction with the IGRA has been frustratingly sparse or non-existent. For contact tracings, the situation is much better, as recent publications from two large studies from Germany and the UK are available [23-25]. However, these studies did not consider the use of a borderline zone. In the German contact tracing study [24], progression to active TB was, however, observed in close contacts with a positive QFT result of between 0.35 and 0.7 IU/mL. Therefore, for these contact tracings, the use of a borderline zone for the QFT is not advisable, either.

For the T-SPOT.TB, a borderline zone has already been proposed by the CDC and ECDC [14,15]. However, as mentioned above, the recommendation is based on little data. As it was shown that the specificity of the T-SPOT. TB is lower than the specificity of the QFT [26], an analogous approach to the QFT might not be justified.

The use of the IGRA for screening close contacts or HCW is endorsed in several national guidelines [15,27-29]. Taking the advantages of the IGRA over the tuberculin skin test into consideration, this is well justified. However, these guidelines lack the recommendation to systematically evaluate the screenings in order to better understand their effectiveness and efficiency and the role of the IGRA within these endeavours. Before consensus on the use of a borderline zone for the QFT can be reached, more and better data on disease prediction with the QFT is needed.

## Competing interest

The authors declare that they do not have any direct or indirect personal relationship, affiliation or association with any party with whom they deal in their day-to-day work that would give rise to any actual or perceived competing interest.

## Authors’ contribution

JTC designed the study, performed the physical examinations and was involved in drafting the paper. AN analysed the data and wrote the first draft of the paper. Both authors read and approved the final manuscript.
